# Direct TAMRA-dUTP labeling of *M. tuberculosis* genes using loop-mediated isothermal amplification (LAMP)

**DOI:** 10.1038/s41598-024-55289-x

**Published:** 2024-03-07

**Authors:** Basma Altattan, Jasmin Ullrich, Emily Mattig, Aline Poppe, Renata Martins, Frank F. Bier

**Affiliations:** 1 Institute for Molecular Diagnostics und Bioanalysis (IMDB), 14476 Potsdam, Germany; 2 Institute for Biochemistry and Biology, University of Potsdam, 14476 Potsdam, Germany; 3 Fraunhofer Institute for Cell Therapy and Immunology-Bioanalytics and Bioprocesses (IZI-BB), 14476 Potsdam, Germany

**Keywords:** Biochemistry, Biological techniques, Biotechnology, Molecular biology

## Abstract

Fluorescent molecule-based direct labeling of amplified DNA is a sensitive method employed across diverse DNA detection and diagnostics systems. However, using pre-labeled primers only allows for the attachment of a single fluorophore to each DNA strand and any modifications of the system are less flexible, requiring new sets of primers. As an alternative, direct labeling of amplified products with modified nucleotides is available, but still poorly characterized. To address these limitations, we sought a direct and adaptable approach to label amplicons produced through Loop-mediated isothermal amplification (LAMP), using labeled nucleotides (dUTPs) rather than primers. The focus of this study was the development and examination of a direct labeling technique of specific genes, including those associated with drug resistance in *Mycobacterium tuberculosis*. We used 5-(3-Aminoallyl)-2′-deoxyuridine-5′triphosphate, tagged with 5/6-TAMRA (TAMRA-dUTP) for labeling LAMP amplicons during the amplification process and characterized amplification and incorporation efficiency. The optimal TAMRA-dUTP concentration was first determined based on amplification efficiency (0.5% to total dNTPs). Higher concentrations of modified nucleotides reduced or completely inhibited the amplification yield. Target size also showed to be determinant to the success of amplification, as longer sequences showed lower amplification rates, thus less TAMRA incorporated amplicons. Finally, we were able to successfully amplify all four *M. tuberculosis* target genes using LAMP and TAMRA-modified dUTPs.

## Introduction

As molecular diagnostics continue to evolve, isothermal amplification methods have emerged in the field of nucleic acid detection with the key advantage of conducting amplification at a constant temperature^1^. These isothermal techniques include, for example, recombinase polymerase amplification (RPA), helicase dependent amplification (HDA), as well as loop-mediated isothermal amplification (LAMP)^2^.

Since its development in 2000^3^, loop-mediated isothermal amplification (LAMP), has been widely implemented as an alternative to conventional Polymerase Chain Reaction (PCR), particularly for point-of-care testing, as it obviates the need for a thermocycler to facilitate denaturation and temperature cycling. LAMP depends on 4–6 primers and a strong strand displacement polymerase to amplify the target DNA sequence, which allows for amplification at high specificity, sensitivity, and rapidity. LAMP has demonstrated the ability to attain comparable or even lower detection thresholds than PCR for specific targets^4^.

Traditional detection methods of LAMP products include visual detection methods, such as turbidimetry using ion indicators, agarose gel electrophoresis and observation of color change in the reaction mixture, with fluorescent intercalating dyes like SYBR Green I^5^. However, these methods are unable to differentiate between amplification of the desired sequence and spurious amplification occurring for example from primer-dimers^6^.

Various detection methods are employed alongside loop-mediated isothermal amplification (LAMP) to identify amplified products. Notably, these methods include coupling LAMP outputs with detection techniques like lateral flow assays (LFA)^7,8^ and microarrays^9^. This application is particularly suited for targeted detection, emphasizing the differentiation of mutations or single-nucleotide differences within LAMP products. Such approaches typically involve pre-labeled primers^10–12^ or probes^12,13^.

An alternative, less common method involves labeling the target during amplification using pre-labeled nucleotides, available with modifications like fluorophores and biotin. Some studies have examined the use of specific modified nucleotides, such as biotin-dUTP^7,9^, Cy5-dUTP^9^, FITC-dUTP^14^, and digoxigenin DIG-dUTP^15^ for direct LAMP labeling. Implementing nucleotide-based labeling can substantially enhance labeling efficiency by increasing the incorporation rates of labeling molecules, thereby increasing assay sensitivity^9,16^, in comparison to pre-labeled primers or probes which result in one label per amplicon. A further added advantage of this approach is its flexibility, as it eliminates the need to pre-label primer sets or probes when altering targets, labeling, or detection protocols^17^.

The incorporation of labeled nucleotides with distinct properties during LAMP has been noted in earlier research to yield differing impacts on both amplification efficiency and the resulting products^9^. This underscores the importance of investigating the influence of diverse modified-dUTP incorporations during a LAMP reaction.

In this context, our study seeks to examine the direct and flexible labeling of LAMP amplicons using 5-(3-Aminoallyl)-2′-deoxyuridine-5′triphosphate, labeled with 5/6-TAMRA (TAMRA-dUTP) during the amplification process. We compare the labeling and amplification efficiency across different *Mycobacterium tuberculosis* genes, including genes associated with drug resistance, by employing real-time LAMP (rtLAMP) and spectrofluorometric analysis.

Tuberculosis (TB) is a bacterial infection caused by *Mycobacterium tuberculosis*. While it is a curable infection, it constitutes a health threat due to the presence of multidrug-resistant strains, which renders it unsusceptible to certain anti-TB agents, making treatment of drug resistant *M. tuberculosis* challenging^18^. Furthermore, the diagnosis of multidrug-resistant tuberculosis is currently conducted through drug sensitivity testing (DST) methods, which are both time-consuming and poorly available in resource-limited settings^19^. Therefore, rapid, and accurate detection of drug resistant *M. tuberculosis* holds vital importance as it addresses a growing global health concern. It is deemed crucial for devising appropriate treatment approaches and plays a pivotal role in curbing its transmission^19,20^.

Drug resistance in *M. tuberculosis* occurs due to genetic mutations, such as single nucleotide polymorphisms (SNPs), insertions, or deletions in specific genes^21,22^. The gene sequences used in this study were 16S rRNA, a highly conserved bacterial sequence that has been widely used for bacterial identification and classification^23–25^; IS*6110*, an insertion sequence found in the *M. tuberculosis* complex species^26^; *katG* and *pncA*, both genes with mutations associated with *M. tuberculosis* resistance against isoniazid (INH) and pyrazinamide (PZA)^27^ drugs, respectively.

## Results

### Finding the appropriate TAMRA-dUTP amount

Various amounts of TAMRA-dUTP were used for LAMP labeling of the 16S rRNA gene, a highly conserved bacterial sequence in *M. tuberculosis* genomic DNA strain. This was done to determine the appropriate TAMRA-dUTP concentration needed for efficient labeling during LAMP amplification. A percentage of 0.5–4% (10–80 µM) of labeled nucleotide and 2 mM unlabeled ones were used.

**Figure 1:**
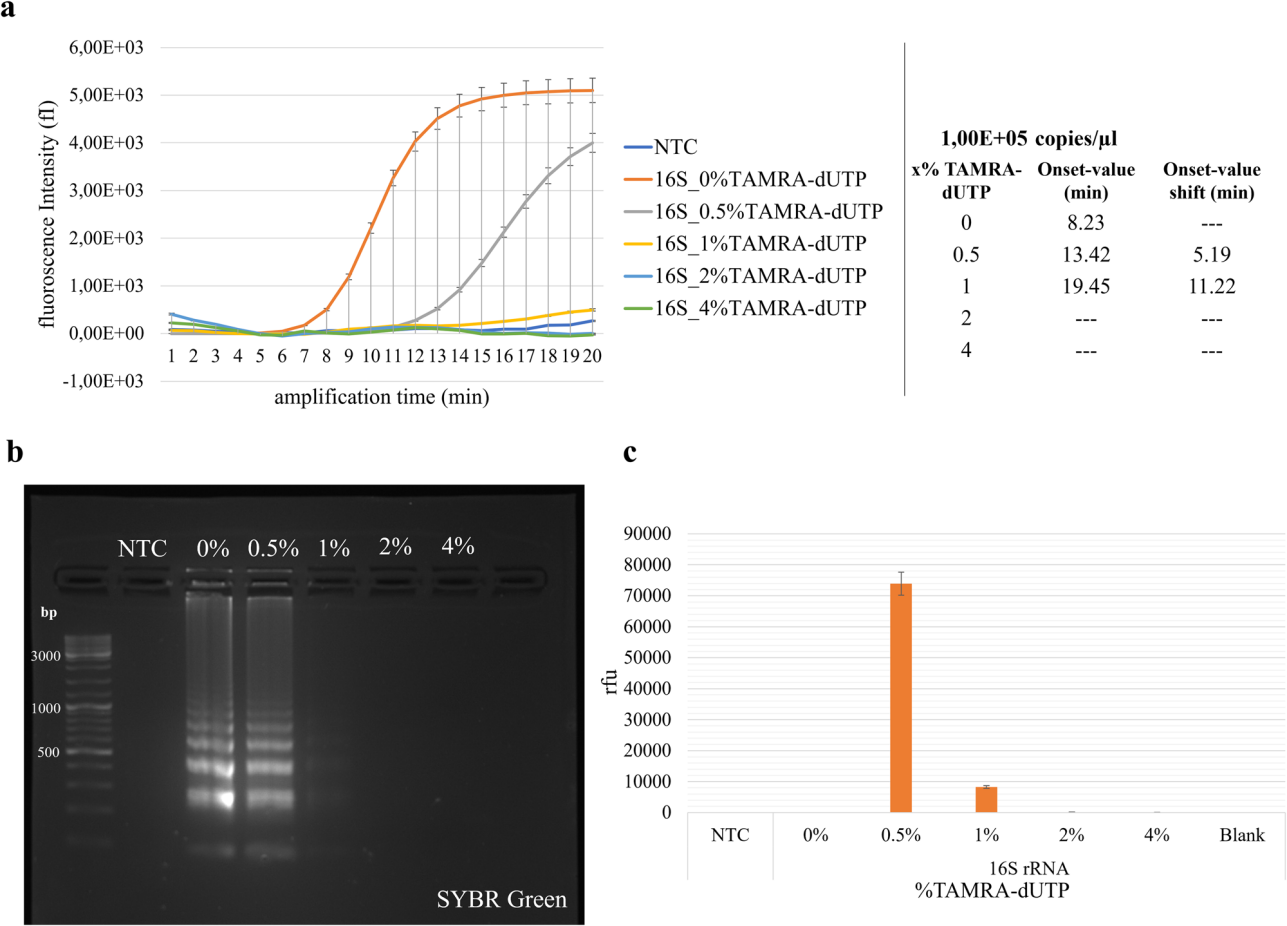
Determination of appropriate TAMRA-dUTP amount. LAMP reactions were performed for 20 min (65°C), using the 16S rRNA gene in *M. tuberculosis* DNA strain as target. (**a**: left) SYBR Green based rtLAMP curves for amplification of 1.00E + 05 copies/µl DNA target per reaction, with various ratios of TAMRA-dUTP (0.5–4%). (NTC: no template control, unlabeled), 0% TAMRA-dUTP: positive control, unlabeled). (**a**: right) Onset-values and corresponding Onset-value shifts performing amplification with unlabeled and x% TAMRA-dUTP-labeled nucleotides. (**b**) Gel electrophoresis and (**c**) graphical visualization of fluorescence signals with purified amplicons after LAMP reaction with different TAMRA-dUTP ratios compared to the unlabeled reaction (*NTC* no template control).

Through SYBR Green based real-time Loop-Mediated Isothermal Amplification (rtLAMP) (Fig. 1a), it was observed that the reactions containing TAMRA-dUTP exhibited a delayed onset of genomic DNA amplification compared to reactions without it. Additionally, this delay was found to be directly proportional to the concentration of labeled nucleotides, with increased concentrations of TAMRA-dUTP leading to further postponements in the initiation of the LAMP reaction. We observed that higher TAMRA concentrations completely inhibited the amplification reaction.

This was shown by the rtLAMP Onset-value shift from 8.23 min for the positive control (no TAMRA-dUTP) and 13.42 min using 0.5% TAMRA-dUTP to 19.45 min using 1% TAMRA-dUTP (Fig. 1a).

Sigmoidal curves could be observed with SYBR green fluorescence intensities, which showed a reduction in amplification efficiency that corresponded directly to the labeled nucleotide concentration. Highest fluorescence intensity was associated with the positive control (no TAMRA-dUTP), with subsequent declines observed in direct response to the increased concentrations of labeled nucleotides used in each LAMP reaction.

Examination of purified amplicons with various concentrations of labeled nucleotides by gel electrophoresis and fluorescence intensity (relative fluorescence units, rfu) revealed a consistent pattern of inhibition in the LAMP reaction using 2% and 4% TAMRA-dUTP (Fig. 1b,c) Conversely, the use of 0.5% TAMRA-dUTP resulted in no observable inhibition of the reaction and a higher fluorescence signal intensity of TAMRA labeled amplicons, while a concentration of 1% TAMRA-dUTP led to the production of only small amounts of target DNA, which was indicated with both the intensity of the bands on the gel and the lower fluorescence signal intensity of the TAMRA incorporated (Fig. 1b,c).

A lower concentration of TAMRA-dUTP (0.375%) was used to test if amplification efficiency could be increased. It was observed that while the reaction started ~ 3.5 min earlier, the fluorescence signal was lower than reactions with 0.5% TAMRA-dUTP, indicating less incorporation per amplified product (data not shown). Thus, based on these results, a concentration of 0.5% (10 µM) TAMRA-dUTP was used for labeling in further experiments.

### Labeling various M. tuberculosis genes

In the second part of the experiments, the method developed with the 16S rRNA gene was applied, using 10 µM (0.5%) TAMRA-dUTP, to test TAMRA incorporation in different *M. tuberculosis* genes, including genes associated with drug resistance.

For this, genomic DNA of three different strains of *M. tuberculosis* were used for the LAMP labeling of 16S rRNA and IS*6110* insertion sequences, as well as the labeling of *katG* and *pncA* genes. The LAMP primers were designed to amplify these genes in both wild-type and resistant *M. tuberculosis* strains.

**Figure 2:**
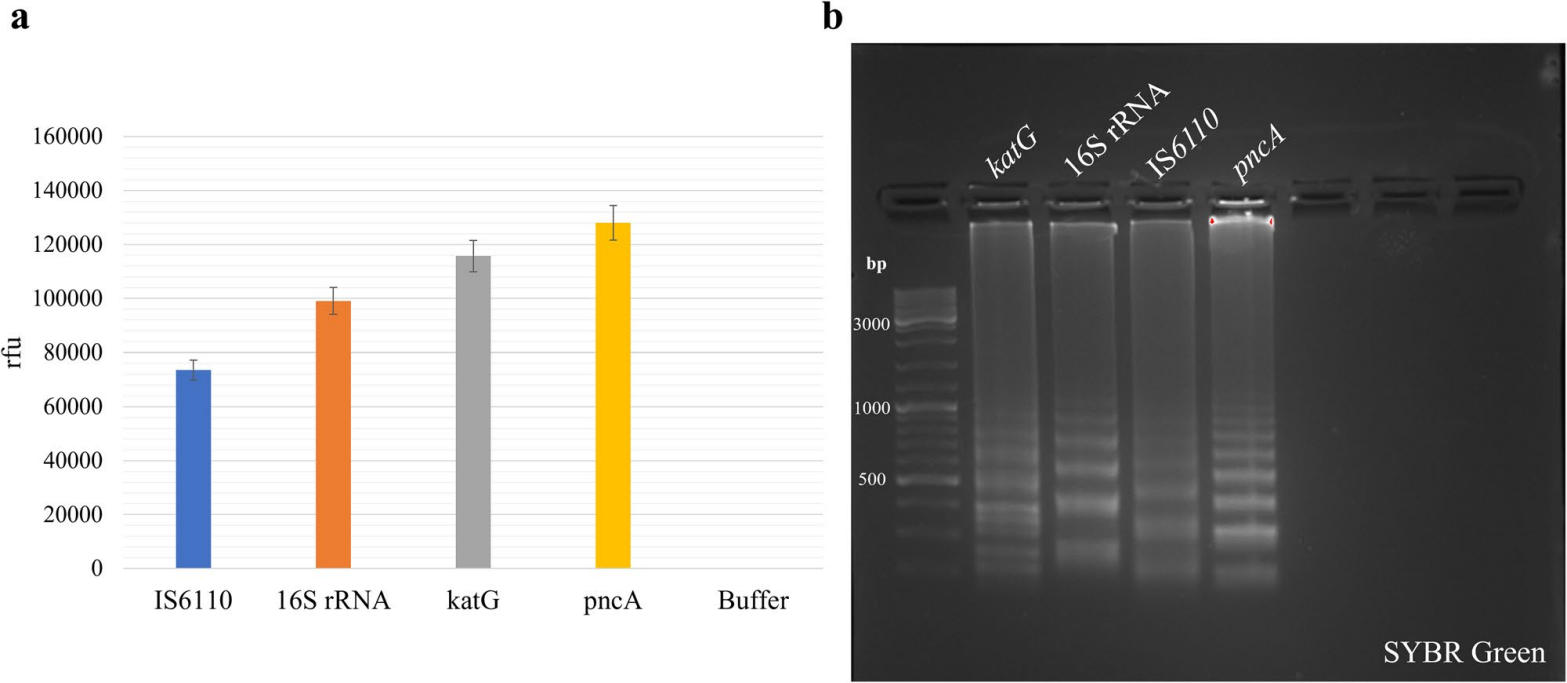
Detection of four different *M. tuberculosis* gene sequences: IS*6110*, 16S rRNA, *katG*, and *pncA*. LAMP reactions were performed for 30 min (65 °C), using 1.00E+05 copies/µl DNA target per reaction, and 0.5% TAMRA-dUTP. (**a**) Graphical visualization of fluorescence signals and (**b**) gel electrophoresis with purified amplicons after LAMP reaction with TAMRA-dUTP.

The fluorescence signal intensities of amplified gene sequences labeled with TAMRA revealed a range in mean intensities. These ranged from approximately 74,000 relative fluorescence units (rfu) for the IS*6110* gene sequence amplicons to 99,000 rfu and 115,000 rfu for the 16S rRNA and *katG* amplicons, respectively, and reached up to 128,000 rfu for the *pncA* sequence amplification product (Fig. 2a). Comparing the fluorescence signal intensities with the length of the fragments produced during LAMP showed that the shorter fragments produced higher signal intensities.

The LAMP products for *pncA* are the shortest with 162 nucleotides (nt) and a percentage of 17.9% dT, followed by the *katG* fragments with 209 nt (15.3% dT) and 16S rRNA fragments with 234 nt (19.7% dT). The IS*6110* amplicons have the longest fragment length with 273 nt (17.6% dT) (Fig. 2b).

**Figure 3:**
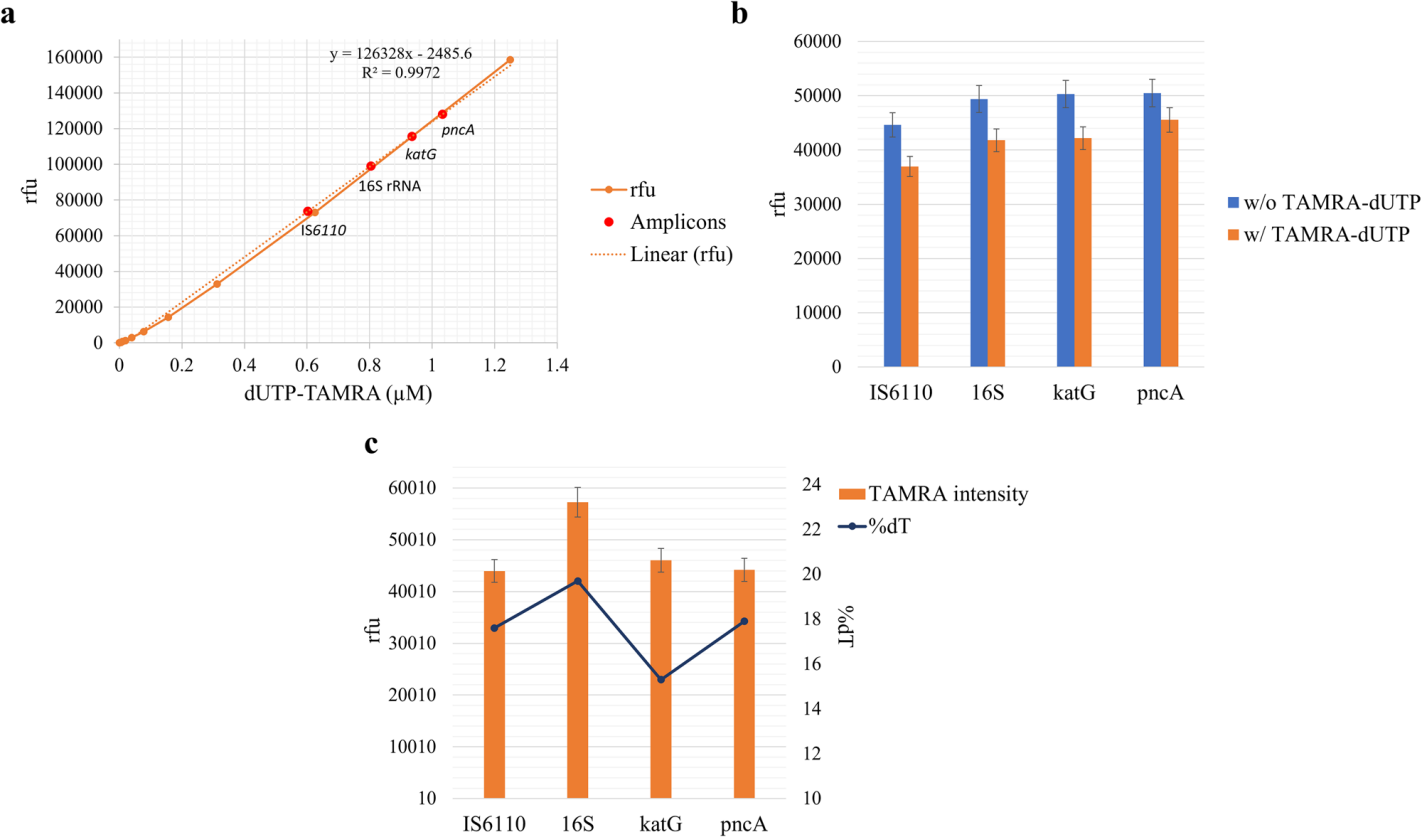
Detection of TAMRA-dUTP in LAMP amplicons. LAMP reactions were performed for 30 min (65 °C), using 1.00E+05 copies/µl DNA target per reaction, and 0.5% TAMRA-dUTP. Incorporation of fluorescent-labeled nucleotides was analyzed by fluorescence spectroscopy. (**a**) Calibration curve for calculation of incorporated TAMRA-dUTP in amplified target DNA samples. (**b**) SYBR Green based graphical representation illustrating the quantity of amplified DNA targets, comparing reactions containing TAMRA-dUTP (w/) to reactions without the inclusion of TAMRA-dUTP (w/o). (**c**) Graphical visualization of TAMRA intensity signals within target amplicons at a concentration of 100 ng/µl, along with the proportion of thymidine (dT) associated with each amplified DNA fragment.

Total TAMRA-dUTP incorporation in each LAMP reaction was quantified using a calibration curve constructed from a serial dilution of free TAMRA-dUTP. Based on the response measurements, the concentrations of TAMRA-dUTP were determined as follows: 1.03 µM in *pncA* amplicons, 0.93 µM in *katG*, 0.80 µM in 16S rRNA, and 0.60 µM in the IS*6110* amplified product (Fig. 3a).

To further characterize the impact of TAMRA-dUTP incorporation on LAMP reactions, we examined the relative quantity of amplicons generated in reactions incorporating labeled nucleotides versus those without any additives. This comparison was facilitated by measuring the signal intensity resulting from SYBR Green intercalation in amplified products. The findings revealed a reduction of 9.8% in *pncA* amplification in reactions containing TAMRA-dUTP. Similarly, amplification of *katG* and 16S rRNA exhibited reductions of 16.2% and 15.4% respectively. Moreover, the analysis determined a decrease of 17.2% in amplification for the IS*6110* target gene (Fig. 3b).

Fluorescence intensities resulting from TAMRA incorporation were measured for LAMP-labeled gene targets standardized to 100 ng/µl. The measured intensities were correlated to the percentage of thymidine (dT) within each amplified DNA sequence. Notably, it was observed that the signal intensities of TAMRA were relatively consistent in the labeled DNA targets. Specifically, the mean intensities measured were approximately 44,000 relative fluorescence units (rfu) for both the amplicons of IS*6110* (273 bp, 17.6% dT) and *pncA* (162 bp, 17.9% dT) gene sequences. Slightly increased signals of 46.000 rfu and 57,000 rfu were measured for the *katG* (209 bp, 15.3% dT) and 16S rRNA (234 bp, 19.7% dT) amplicons, respectively (Fig. 3c).

### Determination of reaction sensitivity

A dilution series of *M. tuberculosis* genomic DNA in a range from 10 to 1.0E+05 DNA copies/µl was used to evaluate the sensitivity of the LAMP labeling reaction. As before, the LAMP was performed by using 0.5% (10 µM) TAMRA-dUTP. The target gene labeled was the 16S rRNA.

**Figure 4:**
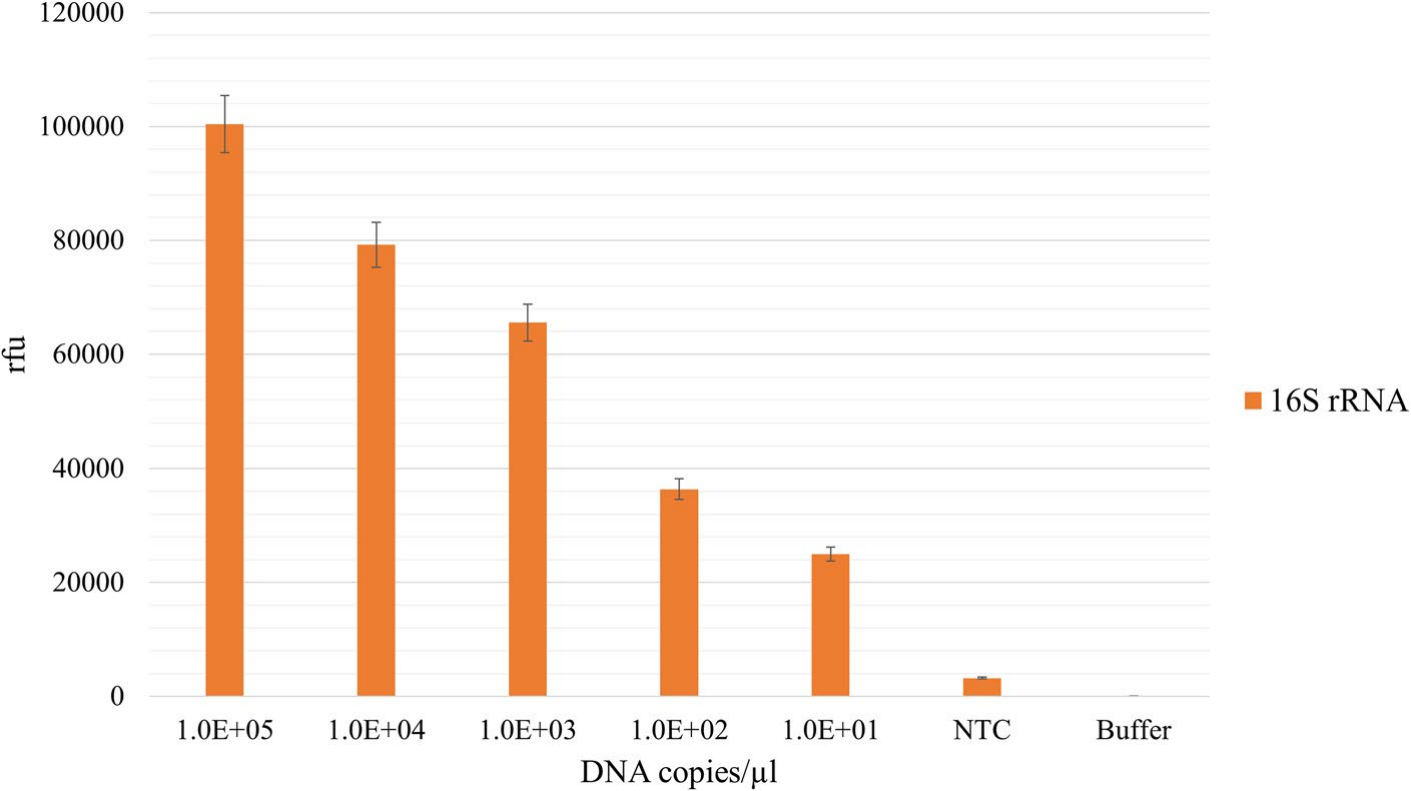
LOD determination for the 16S rRNA gene. LAMP reactions were performed for 30 min (65 °C), using a serial dilution of 10 to 1.00E+05 copies/µl target genomic DNA per reaction, and 0.5% TAMRA-dUTP. Fluorescence intensities were measured of purified amplicons after LAMP reactions with TAMRA-dUTP. (*NTC* no template control; Buffer: 1 × Isothermal buffer).

More than 100,000 rfu were detected for 1.0E+05 DNA copies/µl of the 16S rRNA target. There was a concentration dependent decrease in the fluorescence signal intensity with different amounts of the target genes corresponding to the decreasing number of DNA copies used for the LAMP reactions. At 10 DNA copies/µl the fluorescence intensity was 25,000 rfu for the 16S rRNA amplified product (Fig. 4).

Even with taking the fluorescence of the no template controls (NTC) into consideration, the limit of detection (LOD) for the 16S rRNA gene was as low as 10 DNA copies/µl per reaction.

## Discussion

While the substitution of dTTP with modified dUTP during amplification is still not widely adopted, a handful of studies have employed this method to label products of polymerase chain reaction (PCR)^28,29^, rolling circle amplification (RCA)^16^, recombinase polymerase amplification (RPA)^17,30^, and loop-mediated isothermal amplification (LAMP)^7,9,14,15^ techniques.

Previous studies have demonstrated the modification of amplified products through the utilization of labeled nucleotides. These investigations highlight that the efficiency of amplification, and the incorporation effectiveness are dependent on factors such as the structural features, size, and concentration of the used labeled nucleotides^9,16^.

In this study we showed the successful incorporation of 5-(3-Aminoallyl)-2′-deoxyuridine-5′triphosphate, labeled with 5/6-TAMRA (TAMRA-dUTP) in four different *M. tuberculosis* gene sequences directly during the LAMP reaction. To our knowledge, this is the first time TAMRA-dUTP has been used in direct labeling during the LAMP process.

Performing different detection methods such as rtLAMP, fluorescence spectroscopy and agarose gel electrophoresis, we were able to characterize TAMRA-dUTP incorporation into amplicons during LAMP reaction. Furthermore, the effect of the labeled nucleotides on the LAMP process and the ratio of incorporation were analyzed.

Warmt et al. demonstrated a positive correlation between the fluorescence intensity of the amplicons in LAMP and the increasing concentration of labeled nucleotides. In that study, the quantities of Cy5-dUTP employed per LAMP reaction ranged from 4 (0.28%) to 200 µM (12.5%). Notably, employing 1.5% Cy5-dUTP introduced a 3.5-min delay in the LAMP reaction. The study suggested an optimal ratio of 1–2% of fluorescence-labeled dUTP, striking a balance between fluorescence intensity and the speed of amplification.

Similarly, in this study, the best ratio of labeled nucleotides used in a LAMP reaction was determined to be 10 µM (0.5%) TAMRA-dUTP to 2 mM non-labeled nucleotides. A delay in the onset of the amplification reaction was observed to be an effect of using higher concentrations of labeled nucleotides. An Onset-value shift of more than 11.00 min from the non-labeled LAMP reaction to 1% amount of TAMRA-dUTP per reaction in samples was observed. While using 2% or higher concentrations was seen to inhibit LAMP reaction completely. Based on that, TAMRA-dUTP/dTTP substitution rate was assumed to be 1 per 200 dTTP when using 0.5% labeled dUTP during the LAMP reaction.

Comparable inhibitory effects were observed in rolling circle amplification (RCA) reactions with incorporation of (sulfo-Cy3-dUTP, sulfo-Cy5-dUTP, sulfo-Cy5.5-dUTP, BDP-FL-dUTP, and NH2-dUTP) labeled nucleotides. It was reported that reaction yields decreased with an increasing percentage of labeled nucleotides. Consequently, an optimal percentage of each labeled nucleotide had to be determined to avert the observed reduction in reaction efficiency^16^.

*M. tuberculosis* genome has a high GC content (65.6%)^31^, this is also the case in all genes targeted in this study. Amplifying GC-rich templates are reported to be more challenging compared to non-GC-rich targets due to their high melting temperatures (*T*_m_)^32^, making the GC-rich DNA target, as well as the primers more prone to hairpin or secondary structure formation, thus interfering in the amplification process^33,34^.

Four different gene sequences in *M. tuberculosis* were successfully labeled using TAMRA-dUTP in LAMP. Amplification efficiency and incorporation rate were compared between these four DNA targets. It was observed that the total TAMRA fluorescence intensity depends highly on the length of the amplified fragment. The shortest amplicon (*pncA*, 162 bp) had more than double the fluorescence signal intensity compared to the longest amplified fragment (IS*6110*, 273 bp). To further analyze the total incorporated TAMRA-dUTP in each amplification reaction of the target genes, a calibration curve was constructed from the measurement of the signal intensity of a serial dilution of the free labeled nucleotide. We observed that LAMP reactions of the shortest fragment had 40% more TAMRA-dUTP when compared to the longest fragment. Further results determined that the variation in TAMRA signals stemmed from differing amplification rates associated with the length of the target DNA product.

Similar results have been reported in the literature, evaluating the fragment length in recombinase polymerase amplification (RPA) reactions to fluorescent signal intensity on incorporated Cy5-labeled nucleotides^30^. It was observed that the shortest RPA fragment (141 bp) gave a 20-fold higher fluorescence signal compared to the largest fragment (809 bp).

Furthermore, this study revealed that the incorporation rate of TAMRA-dUTP was relatively consistent regardless of the length of the amplified target gene. However, the length of the amplicon significantly influences amplification efficiency, correlating with the quantity of generated products. Amplification of the DNA target was seen to increase as the fragment length decreased. Consequently, this gives rise to varying quantities of labeled amplicons generated with distinct DNA targets. This was evident through the measurement of TAMRA signal intensities in target DNA products with equal concentrations. Notably, it was seen that there was a slight increase in the signal intensity of the 16S rRNA gene sequence amplicon (Fig. 3c), which could be related to the higher proportion of thymidine (dT) within the DNA sequence, compared to the other DNA targets.

Length of the amplified product can indeed impact both amplification efficiency and the quantity of amplicons produced^35,36^. The effectiveness of LAMP is influenced by the size of the target DNA due to the involvement of strand displacement DNA synthesis as a rate-limiting step in this method. It has been determined that optimal outcomes were achieved with DNA target size smaller than 200 base pairs, while DNA of more than 500 bp amplified, but very poorly^3^.

Additionally, upon conducting a comparative analysis of amplification levels in LAMP reactions that incorporated labeled vs. unlabeled nucleotides, it became evident that the incorporation of TAMRA-dUTP led to an overall reduction in amplification efficiency. This reduction in LAMP products ranged from 9 to 17% when compared with unlabeled reactions. These results reinforce the observed impact of TAMRA-dUTP incorporation in diminishing the efficiency of amplification within LAMP reactions.

The assessment of sensitivity and efficacy of the LAMP reaction while labeling lower DNA copy numbers involved using serial dilutions of *M. tuberculosis* DNA. The results indicated that the labeled amplification reaction remained functional even at a minimum of 10 DNA copies per reaction. This finding underscores that the utilized concentration of 10 µM TAMRA-dUTP was satisfactory for effective labeling of *M. tuberculosis* even at low copy numbers. Importantly, this concentration of labeled nucleotide did not impede the reaction's progression despite the lower amount of target DNA in the reaction.

This study showed the successful integration of the TAMRA fluorophore into four distinct target genes of *M. tuberculosis*. This incorporation was achieved through a direct labeling technique applied during the LAMP process. Moreover, the efficiency of both their amplification and TAMRA integration exhibited variability based on factors such as the length of the amplified product and the concentration of the labeled nucleotide implemented. Furthermore, it was established that TAMRA-dUTP exhibits a consistent incorporation rate throughout LAMP reactions. However, variations in the signal output align with the quantity of generated amplicons, a relationship directly influenced by the length of the DNA fragment.

In conclusion, employing labeled nucleotides for direct amplicon labeling during LAMP reactions provides adaptability and harmonizes with various detection methods like fluorescence spectrometry and microarray technology. This enables simultaneous analysis of multiple LAMP products, eliminating the need for an extensive array of labeled primers and probes, as well as the need for additional post-amplification labeling steps; thus, saving time and resources, and making it suitable for point-of-care settings where rapid and accurate results are essential. Nonetheless, when applying TAMRA-labeled nucleotides in LAMP reactions, certain factors warrant consideration. These factors encompass the effectiveness of the chosen primer set, the length of the target gene arranged for amplification, the elongation time, and the proportion of labeled nucleotides integrated per reaction.

The method described in this study presents a new and efficient strategy to label isothermal amplification products. It is especially promising when used in combination with sequence-specific detection systems. Development of fast, accurate and portable detection platforms of SNPs in genes associated with resistance of *M. tuberculosis* to antibiotics will be key to a more rapid diagnosis and treatment.

## Methods

### LAMP amplification

LAMP was carried out for each strain in a total 25 µl reaction mixture containing 1.6 µM each FIP and BIP, 0.2 µM each F3 and B3, 0.6 µM each FL and BL, 200 µM each dNTP (roboklon, EURx, Cat.No. E0503-01), 0.4 M betaine, 1 × Isothermal buffer (New England Biolabs, B0537), 6 mM MgSO_4_ (New England Biolabs, B1003) and 0.32 units/µl *Bst3.0 polymerase* (New England Biolabs, M0374L). Depending on the experiment the mixture was incubated at 65 °C for an amplification time of 20–30 min, then heated at 85 °C for 3 min to terminate the reaction.

Aminoallyl-dUTP-5/6-TAMRA (Jena Bioscience, NU-803-TAM) were used for labeling of LAMP products with final concentrations ranging from 10 µM to 80 µM (0.5–4%) per reaction. The amount of nuclease free water was adjusted to fit 25 µl reaction volume. Each LAMP reaction contained 1µl of genomic DNA with varied concentrations according to the experiment. For the analysis of LAMP reaction in real-time (rtLAMP) (Analytik Jena, qTOWER^3^G touch thermocycler, Farbmodul 1), 1µl of 50 × SYBR™ Safe DNA Gel Stain (Thermo Fisher Scientific; S33102) was added per reaction.

As part of the experiments, non-templated controls (NTC) were carried out. These controls contained all components of a LAMP reaction except for the inclusion of the genomic DNA template.

### Post-LAMP purification

For the detection and analysis of the LAMP efficiency and TAMRA-dUTP incorporation, via gel electrophoresis, and fluorescence spectroscopy, the products were purified with the GeneMATRIX PCR / DNA Clean-Up Purification Kit (roboklon, Cat.No.E3520) according to the manufacturer’s instructions with a finishing elution step in 25 µl Elution buffer.

### Gel electrophoresis

For gel electrophoretic analysis, 1 µl DNA Gel Loading Dye (6 ×) (Thermo Fisher Scientific; SM0334) was added to 5 µl of the purified samples. For band visualization, a 2% agarose gel was prepared in 1 × TAE buffer (50 × TAE buffer; Jena Bioscience; BU-119–50) containing 1 µl SYBR™ Safe DNA Gel Stain (Thermo Fisher Scientific; S33102) per 50 ml gel to visualize the band. A total volume of 6 µl of the purified and prepared samples were used for electrophoresis which ran at 95 V for 35 min. Detection was done via E-Box gel imaging system (Vilber). GeneRuler DNA Ladder Mixture, Ready to Use (Thermo Fisher Scientific; SM0334) was used for calculation of the fragment length.

### Fluorescence spectroscopy

The fluorescence labeling with TAMRA-dUTP was analyzed with the MARS data analysis software (BMG LABTECH) and FLUOstar Omega microplate reader (BMG LABTECH). Using the fluorescence spectrometer, fluorescence was measured with 15 µl of purified LAMP products and using the following parameters: 544 nm excitation and 590 nm emission filter; gain = 1500. All measurements were conducted as triplicates.

A standard calibration line for the incorporation of TAMRA was created by measuring free TAMRA-dUTP at a dilution range of 10 µM to 4.88 nM.

The evaluation of SYBR Green intercalation was also conducted to perform a relative quantification of amplicons produced within a LAMP reaction. To carry out this analysis, the MARS data analysis software (BMG LABTECH) and the FLUOstar Omega microplate reader (BMG LABTECH) were used. The fluorescence spectrometer was employed to analyze 15 µl of purified LAMP products, using the following settings: an excitation wavelength of 485 nm and an emission wavelength of 520 nm with a gain value of 1000. All measurements were conducted as triplicates.

A baseline signal of 1 × Isothermal buffer (New England Biolabs, B0537) was included in all fluorescence measurements to help detect any intrinsic fluorescence or background noise.

### Concentration measurements

Concentration measurements were conducted using NanoDrop 2000 UV–Vis spectrophotometer (Thermo Fisher Scientific) with 2 µl purified LAMP products.

### Templates and primers

The extracted and purified genomic DNA of *M. tuberculosis* was purchased from the Leibniz Institute DSMZ-German Collection of Microorganisms and Cell Cultures GmbH (DSM 43990). Extracted and purified *M. tuberculosis* mutant strains were purchased from the Institute of Microbiology and Laboratory Medicine—IML red GmbH (IML-00020, IML-02020). IML-00020 strain was used to amplify *katG* gene that contained S315T mutation, while IML-02020 strain was used to amplify *pncA* gene that contained H57D mutation.

Primers were designed with Geneious Prime (Dotmatics). Each reaction contained 6 LAMP-primers specific to the respective *M. tuberculosis* resistance gene (Table 1). Primers were designed based on the genome mutation sequences (provided by Fraunhofer Institute for Cell Therapy and Immunology, Branch Bioanalytics and Bioprocesses IZI-BB). All primers were synthesized and purchased from Biomers.net.

**Table 1:** List of Primers, target genes, and size of amplified fragments.

DNA strain	Target gene	Primer	Sequence (5′–3′)	Fragment (bp)
DSM 43990	IS*6110*	F3	TGTGTGTAGCAGACCTCA	273
		B3	AGGCCGTTGATCGTCTC	
		FIP	CCGCCAGCCCAGGATCCTGCAGGGTTCGCCTACG	
		BIP	AGGGGATCTCAGTACACATCGATCTGCATTGTCATAGGAGCTTCC	
		FL	CGTAGGCGTCGGTGACAAAGG	
		BL	AGGCAGGCATCCAACCGT	
DSM 43990	16S rRNA	F3	ACACATGCAAGTCGAACG	234
		B3	TATTCCCCACTGCTGCCT	
		FIP	TCCCGTGGTCCTATCCGGTATTAGAAGTGGCGAACGGGTGAGTAA	
		BIP	TTGTTGGTGGTGTGACGGCCTAGTATCTCAGTCCCAGTGTGGC	
		FL	AGGCTTATCCCGAAGTGCAGG	
		BL	AAGGAGACGACGGGTAGC	
IML-00020	*katG*	F3	CTTTCGGTAAGACCCATGG	209
		B3	TCCTTGGCGGTGTATTGC	
		FIP	CCGGTGCCATACGAGCTCTTCCAGGCCCGGCCGATCTGGTC	
		BIP	ATGGACGAACACCCCGACGAAATGGAGCAGGGCTCTTCGTCAGC	
		FL	GGAGCAGCCTCGGGTTCGG	
		BL	GATCCTGTACGGCTACGAGTGG	
IML-02020	*pncA*	F3	GTTAACCGGTGGCGC	162
		B3	TAGAACACCGCCTCGATTG	
		FIP	CGGGTCGATGTGGAAGTCCTTGGAGCGACTACCTGGCCGAA	
		BIP	GGACTATTCCTCGTCGTGGCCACCAGACTGGGATGGAAGTCCG	
		FL	CACGACGTGATGGTAGTCCGC	
		BL	GCATTGTGTCAGCGGTACTCC	

The DNA strains refer to the nomenclature of Leibniz Institute DSMZ and Institute of Microbiology and Laboratory Medicine. Primers were synthesized and purchased from Biomers.net.

## Data Availability

The data that support the findings of this study are available from the corresponding author [B.A.] upon reasonable request.
